# Modern Medico-Psychology and Psychiatry

**Published:** 1895-04-06

**Authors:** T. S. Clouston

**Affiliations:** Lecturer on Mental Diseases, Edinburgh University, and Physician Superintendent Royal Asylum, Morningside


### Modern Medico-Psychology and Psychiatry.

By T. S. Clouston, M.D., Lecturer on Mental Diseases, Edinburgh University, and Physician Superintendent Royal Asylum, Morningside.

### CONDITIONS OF MENTAL STUPOR.

Mankind has always been interested in the strange conditions called trance and catalepsy. These are states of mental stupor looked at from the psychological side, but they do not cover all the cases of stupor. They are merely varieties of that disorder—sensational varieties. Stupor is that morbid mental condition in which there are no mental and few bodily responses to outward stimuli, in which the calls of appetite and nature produce no action to gratify them, in which the senses appear to be dormant, though in reality they are not so in most cases. It is a condition of mental negation, with apparent unconsciousness; but, as we shall see, the attention is active in many cases, and the events that seem to pass entirely unheeded are afterwards remembered and spoken of. The patients can commonly stand and walk, and most of them can swallow; in these respects the disease being distinguishable from coma. In addition to these general mental symptoms there are a series of bodily symptoms, the chief of which are a partial vasomotor paralysis of the capillary vessels, so that the hands and feet look dusky, and often swell and are very cold, and sometimes break out into ulcers of the same general nature as chilblains. The face is blank and expressionless, the attitude limp.

The muscles may be in one of three states in different cases. In one set of cases they are flaccid and unresistive, and when a limb is put into a constrained position it will at once fall down to an easy one. The patients will walk when pushed without any resistance, and they will lie down or get up, or eat when food is put into their mouths. In a second series of cases, the muscles are also quite easily moved, and the limbs may be placed in any position you desire, but they remain in that position, not falling down when put up. They are in the so-called "waxy" state. These are the cataleptic cases of stupor. A cataleptic always excites interest and wonder in the popular mind. The expressionless features, the statuesque attitudes, that remain fixed as if a wax figure was being dealt with, the apparent absence of any mind, all give one a strange uncanny feeling about such a case. This state may last for months. You raise one arm and it remains there, you turn the head round and there it is, fixed till turned to a normal position; you raise one finger to the nose, and this meaningless expression of contempt is kept up till the arm falls gradually from unfelt muscular fatigue.

These two varieties, the non-resistive and the cataleptic, together constitute the "anergic" variety of stupor, or the "primary dementia," or the "acute dementia" of the authors. I greatly object to the use of the term dementia to describe any of these cases. I hold that this term should be used only for conditions of mental enfeeblement that are incurable. Stupor I look on as being a distinct variety of mental disease, just as mania or melancholia is. Cases of stupor are curable to the same extent that other forms of recent insanity are curable, that is to the extent of from 50 to 60 per cent. of the cases. They commonly occur in young persons during adolescence, or at all events before 30, it being in fact one form of "adolescent insanity." There is commonly no moral or mental shock or sudden emotional disturbance to cause such cases, though such causes are always associated with them in the popular mind. There are, however, a few cases of stupor from such causes. I saw a man slowly sink into a profound stupor after being ruined by the failure of the City of Glasgow Bank. I saw a young woman who suddenly screamed, became hysterical, and then stuperous on hearing of her lover's desertion of her. In "Called Back" we have a fairly true picture of such a case, making due allowance for the necessities of the novelist, when he has to subordinate disease to art in the picture which he draws. But in far more cases ill-health, disturbances of bodily function, the crises of life or weakened nutrition, are the real causes of the disease, and its treatment largely consists in remedying these disturbances. We commonly use warmth, friction, massage, electricity, baths, nerve tonics, and stimulants, with suitable food, exercise, and moral and mental nursing influences as soon as the mind begins to be in the least responsive. But violent "rousing" measures and sensational stimuli, until the brain condition is improved, often do far more harm than good.

Stupor often occurs as one stage in an attack of mania, and sometimes it is then the prelude to dementia. There is nearly always a nervous heredity in stupor. In these two varieties of stupor the patients sometimes remember all that has taken place around them during their disease, and can describe it more or less accurately afterwards, but commonly the whole time is a blank. The third variety of stupor is that where there is intense resistiveness in the muscles, a sort of motiveless volitional or automatic opposition to any change of posture, to walking, to dressing and to undressing, and to taking food. If you attempt to raise or bend an arm, so much force of resistance is sometimes exercised that it seems as if the bones would break. Such cases are extremely troublesome to manage, and the risk of accidents is great. In these resistive cases there is usually a melancholic element from the beginning. They were often called "melancholia attonita." I class them as cases of melancholic stupor. They often labour under some fearful unexpressed delusion, such as that they are to be burnt alive, that the food offered them is the flesh of their children, that they are to be tortured. Fortunately this variety of stupor is also curable if proper and persistent means are employed. I once had a case of stupor, who for three years never spoke, never ate—having been during all that time fed with the nose-tube—never walked or took any notice of anyone, whose capillary circulation was so weak that his feet and hands were always blue and swollen, and yet he ultimately recovered.

